# Nanomedicine in the Face of Parkinson’s Disease: From Drug Delivery Systems to Nanozymes

**DOI:** 10.3390/cells11213445

**Published:** 2022-10-31

**Authors:** Francisco J. Padilla-Godínez, Leonardo I. Ruiz-Ortega, Magdalena Guerra-Crespo

**Affiliations:** 1 Neurosciences Division, Cell Physiology Institute, National Autonomous University of Mexico, Coyoacan, Mexico City 04510, Mexico; 2 Regenerative Medicine Laboratory, Department of Physiology, Faculty of Medicine, National Autonomous University of Mexico, Coyoacan, Mexico City 04510, Mexico; 3 Institute for Physical Sciences, National Autonomous University of Mexico, Cuernavaca 62210, Mexico; 4 Department of Biological Sciences, Columbia University, New York, NY 10027, USA

**Keywords:** nanomedicine, Parkinson’s disease, controlled drug delivery, nanocarrier, nanozyme

## Abstract

The complexity and overall burden of Parkinson’s disease (PD) require new pharmacological approaches to counteract the symptomatology while reducing the progressive neurodegeneration of affected dopaminergic neurons. Since the pathophysiological signature of PD is characterized by the loss of physiological levels of dopamine (DA) and the misfolding and aggregation of the alpha-synuclein (α-syn) protein, new proposals seek to restore the lost DA and inhibit the progressive damage derived from pathological α-syn and its impact in terms of oxidative stress. In this line, nanomedicine (the medical application of nanotechnology) has achieved significant advances in the development of nanocarriers capable of transporting and delivering basal state DA in a controlled manner in the tissues of interest, as well as highly selective catalytic nanostructures with enzyme-like properties for the elimination of reactive oxygen species (responsible for oxidative stress) and the proteolysis of misfolded proteins. Although some of these proposals remain in their early stages, the deepening of our knowledge concerning the pathological processes of PD and the advances in nanomedicine could endow for the development of potential treatments for this still incurable condition. Therefore, in this paper, we offer: (i) a brief summary of the most recent findings concerning the physiology of motor regulation and (ii) the molecular neuropathological processes associated with PD, together with (iii) a recapitulation of the current progress in controlled DA release by nanocarriers and (iv) the design of nanozymes, catalytic nanostructures with oxidoreductase-, chaperon, and protease-like properties. Finally, we conclude by describing the prospects and knowledge gaps to overcome and consider as research into nanotherapies for PD continues, especially when clinical translations take place.

## 1. Introduction

More than 200 years after James Parkinson’s first trials of “shaking palsy”, most of his original clinical observations remain valid today [1]. Beyond the perception of Parkinson’s disease (PD) as a movement condition (tremor, bradykinesia, rigidity, and others), scientific advances have made it evident that a multitude of non-motor features accompany this condition, such as cognitive impairment, autonomic dysfunction, sleep disorders, depression, and insomnia [2]. The above adds weight to the overall burden associated with PD, the second most common cause of neurodegeneration after Alzheimer’s disease [3]. Throughout its history, significant progress has been made in deciphering the neuropathology of PD and the molecular mechanisms that trigger the pathology and its symptoms, allowing the development of new models and highly effective therapies [4]. These advances have made PD one of the most effectively manageable neurodegenerative diseases, leading to control of symptomatology and improvement of patient’s quality of life through the use of the drug by excellence: levodopa (L-DOPA) [5]. However, like other similar conditions, PD remains a cureless progressive disorder, whose inevitable end is a disability and even death.

Moreover, current approaches to treating symptomatology exhibit long-term side effects that preclude their continued administration or at higher concentrations as the disease progresses. This becomes especially alarming when considering that the leading cause associated with PD is aging [6]. The need for new approaches to slow the progression of the disease is evident. These proposals must be developed primarily as a function of the two pathological features that constitute the PD signature: (i) neuronal loss in the substantia nigra pars compacta (SNpc) with dopaminergic denervation of the striatum, and (ii) intracellular protein misfolding and aggregation into pathological inclusions called Lewy bodies (LBs), the main component of which is alpha-synuclein (α-syn) [7]. Thus, the research on new technologies and potential treatments for PD, as will be described later, should focus either on replenishing the physiological dopamine (DA) conditions necessary for the proper functioning of the nigrostriatal pathway between the SNpc and the striatum (responsible for motor control) or on the prevention and degradation of the protein aggregates that lead to the dramatic neuronal death observed in PD.

In line with the above, breakthroughs have been achieved recently. In particular, nanomedicine, the medical application of nanotechnology [8], has made substantial progress in controlled drug delivery and treatment platforms with promising results. The above, refocused on the pathophysiology of PD, could represent an outstanding tool for optimizing the medicinal effectiveness of anti-Parkinson drugs and even generate new approaches from more physiological and compelling perspectives, thus reducing the side effects associated with traditional treatments. Therefore, throughout this review, we comprehensively describe the most promising nanostructures developed and evaluated as potential new treatments for PD. Firstly, we briefly cover our current knowledge of PD neuropathology to better comprehend the biochemical and molecular bases that must be considered when new therapies are developed, especially those related to DA depletion and protein misfolding and aggregation. Subsequently, we describe the most significant advances in the application of nanomedicine in controlled drug delivery, protein aggregation inhibition, and selective proteolysis focused on PD, as well as a description of potential strategies that could be followed to optimize biocompatibility, selectivity, and efficacy, as well as to achieve non-invasive administration routes. Finally, we offer a concise review of the main limitations and safety concerns to consider when developing nanomedicine-based therapies, especially in the areas of surface functionalization and nanotoxicity, since only by filling these gaps in knowledge will research be able to enable clinical translation. The aforementioned is intended to expand our understanding of the mechanisms involved in PD and the actual and potential perspectives that nanoparticles (NPs) offer so that researchers might be capable of developing better procedures to treat the symptomatology, slow the progression of the disease, and, potentially, advance in the search for a cure.

## 2. Current Knowledge of Parkinson’s Disease’s Neuropathology

### 2.1. Normal Function of the Dopaminergic Neurons

#### 2.1.1. Neurophysiology of Motor Control

The basal ganglia perform the coordination and execution of activities requiring motor, cognitive, and limbic circuitry in concert with the cortex [9]. The striatum, the main input structure of the mammalian subcortical basal ganglia, is abundantly innervated by projections from the cortex, thalamus, and substantia nigra. The latter interaction is termed the nigrostriatal dopaminergic pathway, a crucial structure in the modulation of a wide range of behaviours, from learning and working memory to motor control by DA neurotransmission [9]. The substantia nigra is a flattened oval structure located on the dorsal aspect of the cerebral peduncle, with approximately 220,000 dopaminergic neurons in each hemisphere [10,11]. Among its component regions, the substantia nigra pars compacta (SNpc) consist mainly of dopaminergic cells that project massively to the dorsal striatum [12]. These neurons are further subdivided according to their chemical properties in dorsal and ventral tiers, which project to different striatal compartments and show varied vulnerabilities to degeneration in PD, the latter being the most susceptible [13].

The nigrostriatal pathway exerts its regulation on motor behaviours through somatodendritic DA release in the SNpc and axonal DA release in the striatum [14]. This neurotransmission is regulated at various levels (synthesis, vesicular transport, and uptake of DA) and by a variety of factors; Sulzer et al. (2016) [15] provided a detailed review of these processes. Below, we highlight the critical points to consider. First, the mechanism begins with the synthesis of the to-be-delivered DA from tyrosine in the perikaryon (a process regulated by the activity of tyrosine hydroxylase, TH [16]) and its transport by the vesicular monoamine transporter 2 (VMAT2) into small (~40–50 nm diameter) synaptic vesicles for storage and protection from the metabolic breakdown [17,18]. This procedure keeps free-cytosolic DA concentrations at a minimum [19]. Such regulation is of vital importance due to the high susceptibility of DA to oxidation under cytosolic conditions, which leads to the formation of neurotoxic species [20,21].

The number of vesicles in the synapsis depends on Ca^2+^ homeostasis and presynaptic proteins, including synapsins, tomosyns, and α-syn [22]. Furthermore, autoreceptors and heteroreceptors (glutamate, GABA, acetylcholine) can modulate axonal DA release [23]. When neurotransmission occurs, synaptic vesicles fuse with the axon membrane in two possible states, reversible and full fusion [24], and liberate DA in quantal sizes, with contents of ~33,000 DA molecules per axonal vesicle [25]. The cross-synaptic diameter of a striatal dopaminergic synapse is 0.3–0.6 μm [26], whereas DA can diffuse up to ~1 μm away from the release site [27]; similarly, anatomical data suggest that, on average, one DA synapse per 20 μm^3^ takes place in the striatum [28]. Such conditions in the spheres of influence of DA readily derive into synaptic “spillover”, with gradients of efficacy that depend on receptor sensitivity [27,29].

Among the family of five DA receptors present in the striatum, receptors 1 and 2 (D1R and D2R, respectively) are responsible for generating the signalling cascades that regulate motor control [30]. Upon excitation, D1Rs in the putamen follow a direct pathway in which an inhibitory action is generated on the internal globus pallidus, thereby interrupting its inhibition on the ventral anterior nucleus of the thalamus, allowing the latter to promote movement in the motor cortex. On the contrary, upon neurotransmission, the D2Rs in the putamen activate an indirect pathway in which the inhibition on the external globus pallidus is interrupted, triggering its inhibitory response on the subthalamic nucleus, which prevents it from exciting the internal globus pallidus and thus continuing the direct pathway as described above.

Once the postsynaptic neuron generates action potentials as a result of the synapse, DA molecules are rapidly released from the receptors contributing to the extracellular concentration of the neurotransmitter. Given DA susceptibility to oxidation, extracellular DA concentration must remain low (10–20 nM) under resting conditions [31]. For this purpose, dopaminergic neurons express DA transporters (DAT) in extrasynaptic and synaptic sites, which reuptake released DA contributing to extracellular clearance [32,33]. Nonetheless, DA diffusion away from a synapse follows such a rapid time course that the cloud of released DA, diffusing in three dimensions, encounters predominantly extrasynaptic DA autoreceptors on DA axons and heteroreceptors on neighbouring cells, including medium spiny neurons [34]. Therefore, released DA will be taken up only when it encounters DATs on dopaminergic axons. Non-reuptaken DA will follow degradation by catechol o-methyltransferase and presynaptic monoamine oxidases, whose oxidation by-product, H_2_O_2_, can cause oxidative stress and, hence, neurotoxicity [35].

**Figure 1:**
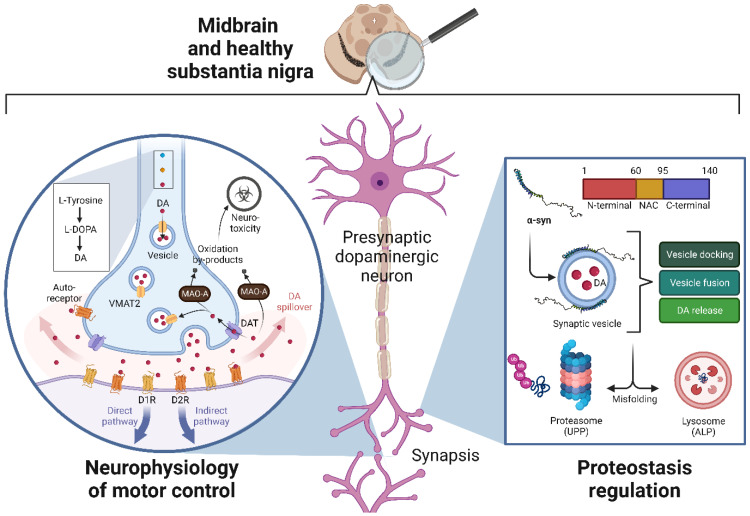
Neurophysiology of motor control (**left**) and proteostasis regulation (**right**) in healthy midbrain substantia nigra pars compacta dopaminergic neurons. In regular motor control, DA (synthesized from L-tyrosine) is encapsulated in synaptic vesicles by VMAT2. and then released to excite DA receptors D1R and D2R, thereby triggering the direct and indirect pathways of motor regulation, respectively. DA can spillover if not reuptaken by the neuron through the DAT. Free DA in the cytosol and the extracellular matrix can be oxidized by MAO-A into neurotoxic by-products. On the other hand, α-syn regulates the docking, fusion, and release of DA from synaptic vesicles are regulated. When the protein is misfolded, dopaminergic neurons eliminate it through the UPP or the ALP pathways. DA: dopamine; L-DOPA: levodopa; VMAT2: vesicular monoamine transporter 2; MAO-A: monoamine oxidase A; D1R: dopamine 1 receptor; D2R: dopamine 2 receptor; DAT: dopamine transporter; α-syn: alpha-synuclein; NAC: non-amyloidogenic component; Ub: ubiquitin; UPP: ubiquitin-proteasome pathway; ALP: autophagy-lysosomal pathway. Figure made in BioRender.com.

Figure 1 (left side) shows a simplified model of DA neurophysiology, from synthesis to reuptake after synapse. This highly regulated process can undergo alterations at different levels of regulation, which can lead to pathophysiological processes, as described below.

#### 2.1.2. Physiological Regulation of Proteostasis and Alpha-Synuclein

Because the dynamic regulation of a balanced and functional proteome (proteostasis) is essential for dopaminergic neurons’ normal functioning, the correct operation of their protein degradation systems is critical for controlling protein quality and eliminating proteins that have been damaged or misfolded. The two central protein degradation systems in dopaminergic neurons are the ubiquitin-proteasome pathway (UPP) and the autophagy-lysosomal pathway (ALP) [36]. In the UPP, proteasomes degrade short-lived proteins tagged with ubiquitin molecules [37]. In contrast, ALP is responsible for degrading long-lived proteins, cellular components, and organelles through the lysosomal compartment, with a dual purpose: to remove harmful intracellular components and to recycle macromolecules and organelle proteins to ensure the renewal of the proteome [38]. Together, both mechanisms maintain protein homeostasis within the neuron. Such is their importance that alterations in their function can result in the accumulation of misfolded proteins and the development of neurotoxicity. Among the riskiest proteins in terms of aggregation in dopaminergic neurons, alpha-synuclein (α-syn) stands out above all others.

The α-syn is a small protein (14 kDa, 140 amino acids) highly expressed in neurons [39] and composed of three domains: (a) an N-terminal lipid-binding alpha-helix; (b) a non-amyloid-component (NAC); and (c) a C-terminal acidic tail [40]. Widely regarded as an intrinsically unfolded monomeric protein in the cytosol, α-syn is involved in synaptic activity through the regulation of vesicle docking, fusion, and neurotransmitter release [41]. Indeed, Zaltieri et al. (2015) [42] observed that the absence of α-syn decreases the amount of membrane DAT and increases the density of synapsin III (a protein that negatively modulates DA release in nigrostriatal neurons) in presynaptic boutons, thus altering the proper clustering of synaptic vesicles at the active zone; this coincides with a reduction of DA release. Similarly, aggregation of α-syn could change the rearrangement of DA terminals, similar to its absence. The predominantly unstructured conformation of α-syn makes it susceptible to several posttranslational modifications, such as phosphorylation [43]. In fact, phosphorylation of Serine 129 has been associated with an increased propensity of aggregate formation due to misfolding [44]. As will be described below, the accumulation of α-syn into prefibrillar forms, and then its assembly into higher molecular weight aggregates (synucleinopathy [45]), induces cellular toxicity through synaptic vesicle impairment, mitochondrial dysfunction, generation of oxidative stress, and endoplasmic reticulum stress, suggesting that they are the most significant contributors to pathogenesis in PD [46].

Although both UPP and ALP are responsible for degrading misfolded α-syn (Figure 1, right side), they are also affected in synucleinopathies. We will briefly describe the precise mechanisms in the next section.

### 2.2. Pathophysiological Mechanisms of Neurodegeneration in PD

#### 2.2.1. Loss of Proteostasis

##### Alpha-Synuclein Misfolding, Aggregation, and Propagation

Dopaminergic neurons are particularly susceptible to α-syn aggregation, primarily due to three reasons: (i) their high metabolic rate, which involves high rates of oxidative phosphorylation and ROS generation that oxidizes proteins, prompting them to aggregation [47,48]; (ii) their inability to divide and, thus, dilute away protein aggregates by stealthily passing them to daughter cells, hence leading to accumulation of aggregates and sequestering of other proteins [49]; and (iii) the rapid neurotransmitter release involving thousands of events occurring per minute, numerous proteins, and protein conformational modifications, which can lead to chaperone machine failure, and an inefficient neurotransmitter release [50].

**Figure 2:**
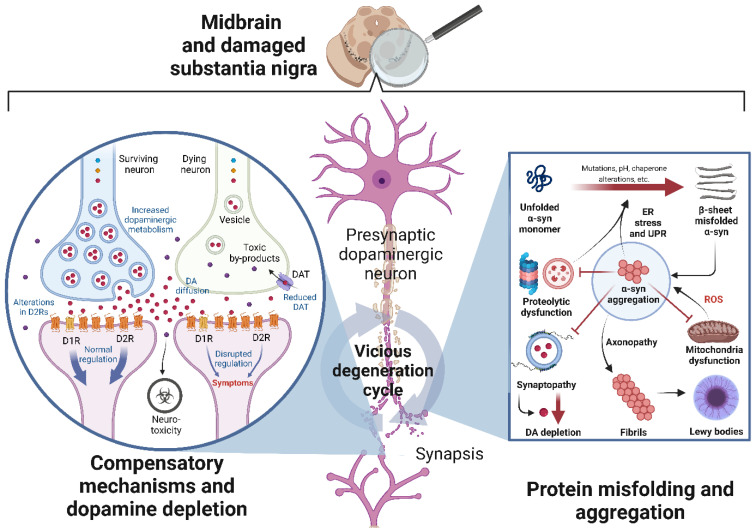
Pathophysiological mechanisms of neurodegeneration: dopamine depletion (**left**) and loss of proteostasis (**right**) in damaged midbrain substantia nigra pars compacta dopaminergic neurons. Upon dopaminergic neurodegeneration, surviving neurons activate compensatory mechanisms to maintain striatal DA concentrations and keep normal motor regulation. Such mechanisms include increased dopaminergic metabolism, alterations in D2Rs, reduced DAT expression, and an increment in DA diffusion. Nonetheless, if neurodegeneration continues, DA depletion translates into a disrupted regulation of motor control and, hence, symptoms. Such neurodegeneration is caused, among other factors, due to the loss of proteostasis. When misfolded into a β-sheet composition due to mutations, pH, or chaperon alterations, α-syn can aggregate. This aggregation disrupts the UPP and ALP proteolytic pathways (inhibiting misfolded α-syn degradation); stresses the ER activating the UPR; causes mitochondrial dysfunction, which generates toxic ROS species that further facilitate α-syn aggregation; alters normal synaptic processes (synaptopathy), which in turn causes a DA depletion; and creates fibrils that travel through axons (axonopathy) into the cytoplasm, where fibrils sequester organelles and proteins to form LBs. Consequently, α-syn misfolding and aggregation generate neurotoxicity in dopaminergic neurons, causing the vicious degeneration cycle observed in PD. DA: dopamine; D1R: dopamine 1 receptor; D2R: dopamine 2 receptor; DAT: dopamine transporter; α-syn: alpha-synuclein; ER: endoplasmic reticulum; UPR: unfolded protein response; UPP: ubiquitin-proteasome pathway; ALP: autophagy-lysosomal pathway; ROS: reactive oxygen species; PD: Parkinson’s disease. Figure made in BioRender.com.

Environmental and intrinsic factors, such as mutations, pH, and chaperone alterations can induce α-syn to form fibrils by converting either all or part of the previously unstructured polypeptide into well-defined β-sheet-rich secondary structures (Figure 2, right side) [51]. Such misfolding increases their propensity to aggregate with other misfolded proteins, leading to fibrillation, aggregate formation, protein and organelle sequestering, and, ultimately, Lewy body (LB) formation; for a more detailed description of the mechanisms of α-syn aggregation, see Mehra et al. (2019) [52]. Although known to localize in presynaptic terminals, the oligomers and aggregates can be found in cell bodies and neurites, which indicates a widespread toxic action [36]. Indeed, the aggregation dynamic is accentuated due to the prion-like nature of the misfolded α-syn, which allows it to self-propagate and spread progressively between interconnected brain regions through a cell-to-cell transmission mechanism [43,53,54].

##### Proteolytic Dysfunction, Endoplasmic Reticulum Stress, and the Unfolded Protein Response

Although, as mentioned above, the main mechanisms responsible for the degradation of misfolded α-syn are the UPP and ALP, the progressive accumulation of α-syn leads to a disruption of these systems. It has been shown that the protein can inhibit certain enzymatic activity domains in proteosomes [55] by direct binding to the S60 or Rpt5 subunits of the 19S proteasome or the β5 subunit of the 20S proteasome [56,57]. Similarly, aggregated α-syn can bind to lysosomal membrane proteins (such as Lamp-2a), blocking their function [58]. Furthermore, α-syn also inhibits the expression of proteins necessary for the assembly of autophagosomes, impairing macroautophagy [59]. Thus, loss of proteostasis regulatory mechanisms in dopaminergic neurons leads to the aggregation of α-syn, whose misfolded form, in turn, leads to the disruption of protein cleavage mechanisms, creating a vicious circle.

As a result of cellular proteostasis dysfunction, the endoplasmic reticulum (ER), responsible for protein synthesis, post-translational processing, and protein folding, becomes stressed, and several intracellular signal transduction pathways activate in an attempt to restore ER homeostasis. These processes are collectively referred to as the unfolded protein response (UPR) [36]. Nonetheless, when the capacity of UPR to maintain proteostasis is overwhelmed, cells activate the control of cell death by apoptosis [60]. In this regard, α-syn overexpression correlates with chronic activation of multiple pathways of the UPR system and ER stress-mediated apoptosis [61], ultimately leading to inflammation and neurodegeneration. Indeed, ER dysfunction has been positioned as an early component of PD pathogenesis [62,63].

Thus, taken together, misfolding and aggregation of α-syn (due to the reasons described above), destabilization of the physiological mechanisms for aggregate clearance, and subsequent activation of ER stress and UPR result in an aporia responsible for the increase in toxic α-syn (synucleinopathy), as well as the emergence of pathological processes responsible for neuronal dysfunction, and, ultimately, neuronal death and the symptomatology of PD.

##### Consequences of Alpha-Synuclein Misfolding and Aggregation

Synaptopathy is the first consequence of α-syn misfolding and aggregation [64]. As mentioned above, under normal conditions, α-syn in axonal terminals is tightly bound to the membrane on synaptic vesicles [65]. When in the form of oligomers and protofibrils, accumulated α-syn impairs synaptic vesicle pools and changes the distribution of proteins in the presynaptic complex, subsequently altering proteins in the presynaptic complex, with the consequent disruption of the synaptic vesicles in the active zone [66,67]. Furthermore, synaptopathy leads to axonopathy, as α-syn deposits migrate through axons into the neuronal cytoplasm [68], where aggregates are sequestered in LBs. In addition, α-syn is known to dysregulate mitochondrial function, a key element in the pathogenesis of idiopathic and familial PD [69]. Indeed, accumulation of α-syn inside these organelles leads to damage of complex-I activity and mitochondrial receptors [70,71], ultimately resulting in the impairment of the mitochondrial protein import machinery, reduced respiration, and excessive production of reactive oxygen species (ROS), which also increases Ca^2+^ levels [72]. Together, these phenomena, in turn, favour the further α-syn misfolding and aggregation, eventually creating a potential death spiral [73]. The result, in either scenario, is neuronal death, both by toxic factors generated as a result of the cascade initiated by synucleinopathy and from the release of cytochrome c and other pro-apoptotic factors caused by mitochondrial destabilization [69]. The loss of dopaminergic neurons and their projections translates directly into depletion of physiological DA levels, as will be discussed in the next section.

#### 2.2.2. Loss of Dopaminergic Neurons and Their Projections

##### Compensatory Mechanisms upon Dopaminergic Neuron Death

Upon nigrostriatal lesions, surviving dopaminergic neurons undergo functional changes to preserve DA availability in the striatum (Figure 2, left side) [74]. The traditional first response is increased dopaminergic metabolism, in which remaining dopaminergic neurons make an effort to synthesize bigger concentrations of DA to compensate for the depletion due to the loss of their partners [75,76,77]. Simultaneously, D2-type DA receptors density increases in striatal neurons [78,79], while DAT expression is reduced to allow for extracellular DA concentrations to remain physiological [80,81]. This last mechanism raises, in turn, the extrasynaptic diffusion capacity of normally limited DA [82,83]. In this way, due to the profuse axonal synaptic arborization in the nigrostriatal projections, DA released from a few hundred neurons coincide on a single striatal site making the impact of early nigrostriatal loss negligible [84,85]. Finally, although several more changes have been identified, such as modifications in the serotoninergic system, the medium spiny neurons, an increment in TH expression in the striatum, and electrophysiological changes, their pathophysiological significance has not been defined as compensatory or the consequence of DA depletion [86].

This compensatory response allows a buffering of the progression of symptomatology in the early stages of PD; however, the advancement of neurodegeneration renders these mechanisms insufficient, leading to an increment depletion of DA and, therefore, to the dysregulation of motor activity, as will be described below.

##### Consequences of Dopamine Depletion in the Dorsal Striatum

DA being the modulator of the motor circuitry through its interaction with the basal ganglia, it is evident that depletion in its physiological levels will play an essential role in the motor pathophysiology of PD. Indeed, bradykinesia, the core parkinsonian symptom of PD comprising slowness of movement, is the clinical hallmark that most closely correlates with DA deficiency [87]. It is characterized as a low vigour to move in a DA-depleted state; interestingly, it responds well to DA replacement therapy [88]. Nonetheless, upon DA replete, several patients exhibit a seemingly opposite behaviour: purposeless movements, a phenomenon termed dyskinesia [89]. In terms of pathophysiology, this dyskinetic response is driven by the progressive failure of cellular reuptake and recycling of striatal DA, leading to fluctuating DA levels [90,91]. In this sense, as opposed to bradykinesia, dyskinesias are due to excessive enforcement of movement vigour in the DA repleted state [92,93,94]. This way, a dopaminergic restitution therapy focused on the nigro-dorsal striatal circuit should continuously compensate for the lost DA, preventing fluctuations.

##### Consequences of Dopamine Depletion in the Ventral Striatum

**Figure 3:**
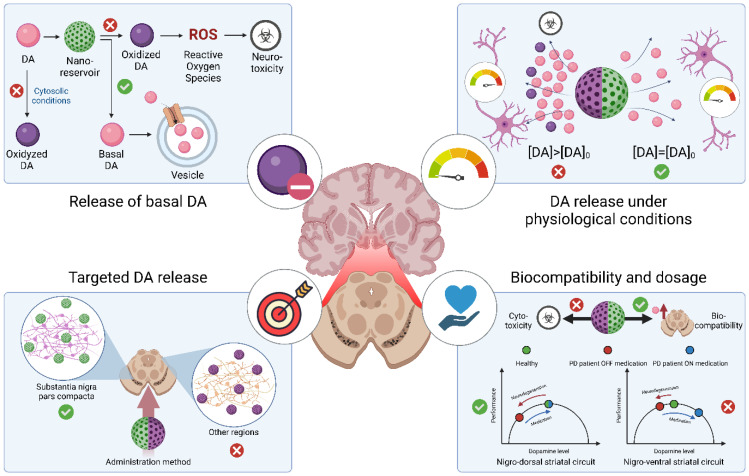
Conditions required for the development of novel nanostructure dopamine delivery systems. Firstly, the nanoreservoir must stabilize DA in its basal state, avoiding its oxidation until its release and encapsulation in synaptic vesicles since oxidized DA derivatives increase the production of ROS, which generates neurotoxicity. In addition, the release rates of stabilized DA must meet the physiological needs of dopaminergic neurons without exceeding physiological extracellular [DA]_0_ concentrations. It must also be ensured that such release takes place only in the necessary regions, regardless of the method of administration, so that DA is not wasted or oxidized, causing neurotoxicity. Finally, the nanoreservoir must comply with the principle of biocompatibility and adequate dosage since an overdose of DA could lead to dysregulation of motor performance. DA: dopamine; ROS: reactive oxygen species. Figure made in BioRender.com.

However, the dopaminergic compensation necessary for the nigrostriatal pathway will not necessarily be correct for the other dopaminergic pathways. Along with the SNpc, there is another midbrain dopaminergic neuron nucleus located in the ventral tegmental area (VTA), which project mainly to the nucleus accumbens (ventral striatum, mesolimbic pathway), and the prefrontal cortex (mesocortical pathway) [95,96]. Although to a lesser extent than in SNpc, the VTA also undergoes neurodegeneration in PD [97,98], which creates functionally relevant changes affecting its core functions in reward, prediction error coding during learning [95,99], risk assessment, effort evaluation, and motivational drive [100,101]. The difference in dopaminergic neuronal death rates between SNpc and VTA results in what is known as the “overdosing theory” when DA restitution therapies are administered [95]. This is due to the nature of the relationship between the level of striatal dopaminergic innervation and optimal neural processing in the striatum, which is characterized by an inverted U-shaped Yerkes-Dodson function (Figure 3) [102]. Such a relationship indicates an optimal point between DA levels and performance (motor or cognitive); when neurodegeneration occurs, the balance shifts, and dopaminergic restitution allows a return to equilibrium. Nonetheless, the dosage required for an optimum in SNpc will cause relative “overdosing” in the VTA, rightward shifting towards the descending part of the U-shaped curve, and causing suboptimal circuit function with adverse effects on cognition (refer to the work of Cools and coworkers [103,104]). Thus, apart from continuously compensating DA loss, DA restitution therapies must ensure selective liberation of required concentrations in the different dopaminergic nuclei.

## 3. Controlled Drug Delivery on Parkinson’s Disease

### 3.1. Dopamine Administration as A Physiological Approach

Advances in the controlled release of L-DOPA, as well as adjuvant administration approaches that reduce its side effects, suggest the possibility of developing more effective treatments for PD. However, research to reverse DA depletion could take a more straightforward approach, focusing on normal physiological processes rather than pathological ones, similar to models in other fields [105,106]. In this regard, recent research has focused on the release not of precursors but of DA itself, which is ultimately the molecule necessary for dopaminergic stimulation.

However, unlike L-DOPA, DA presents several problems related to its stabilization and release in vivo. In the first instance, DA is a highly reactive molecule. Free cytosolic DA is susceptible to oxidizing into dopamine-*o*-quinone and aminochrome due to the dissociation of the protons in their hydroxyl groups: these reactions result in the formation of superoxide radicals (O_2_^−^) [107]. Such oxidation plays a major role in the neurodegenerative processes of PD as these oxidative products induce mitochondria dysfunction by mitochondrial membrane depolarization, reduction of ATP synthesis, α-syn accumulation into neurotoxic protofibrils, proteasomal and lysosomal dysfunction, and oxidative stress [21,108,109]. Thus, the administration of DA requires delivery systems that allow the preservation of the basal state of the molecule, especially under physiological pH conditions. In addition, the release should result in its uptake through monoaminergic synaptic vesicles, which have a relatively low pH (2 to 2.4 pH units lower than the cytosol), keeping DA from becoming oxidized [17]. Furthermore, in addition to being conserved in its basal state, DA must be released in a controlled manner allowing its use according to the needs of the dopaminergic neurons without causing an overdose. In this sense, the release system must maintain physiological extracellular concentrations of DA (~23.2 μM in the cortex and ~332.6 μM in the striatum, according to Zhang et al. (2018) [110]), and endow its utilization primarily in the SNpc, the region previously described as mainly affected in PD and where the nigrostriatal pathway responsible for fine motor control begins [111].

In summary, DA delivery systems must meet three main conditions to represent a viable option: (1) to allow stabilization of DA in its basal state, (2) to facilitate a release under physiological conditions, and (3) that this release takes place in the necessary regions, particularly in the SNpc. In addition, (4) the administration of these systems must not generate cytotoxicity or dosage-related side effects (as mentioned before for the “overdosing theory” when DA is restituted in SNpc and VTA), so biocompatibility and dosage must also be critical parameters in developing these structures (a summary of these requirements is offered in Figure 3). In recent years, new nanostructured devices for controlled DA release have been developed, which could represent alternatives to current therapies for PD symptomatology. The main advances are described in the following section.

### 3.2. Nanocarriers for Controlled Release of Dopamine

**Figure 4:**
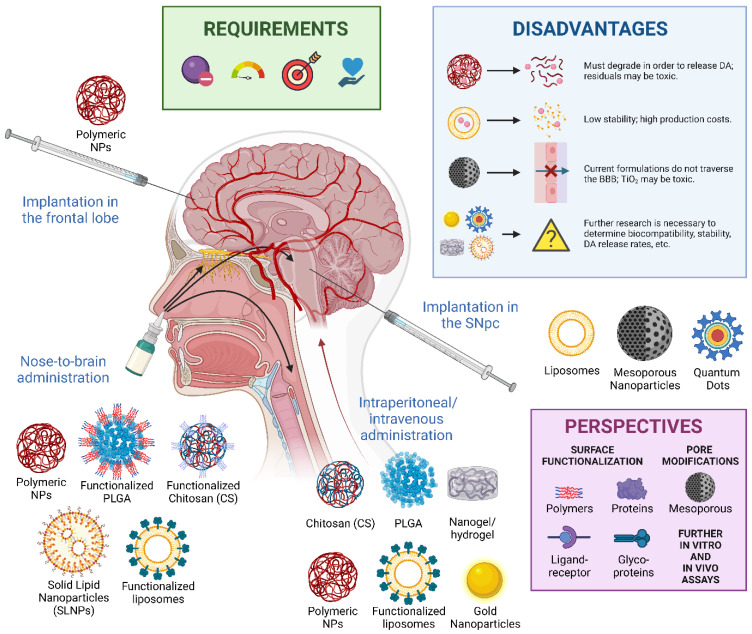
Current research on nanocarriers for controlled release of dopamine. The new nanocomposites for controlled release of DA are governed under the requirements described above: preservations of the basal state of DA, release at physiological concentrations and in the regions that require it, and preservation of biocompatibility without causing side effects. The different types of compounds have been developed following different routes of administration and have certain disadvantages depending on the type of structure. The main perspectives are based on surface functionalization to improve cytocompatibility and non-invasive routes of administration, as well as on modifying the porous structures to increase the concentration of stabilizable DA, as well as the implementation of further in vitro and in vivo studies. DA: dopamine; NPS: NPs; PLGA: poly(lactic-co-glycolic acid); CS: chitosan; SLNPs: solid lipid NPs; SNpc: substantia nigra pars compacta; TiO_2_: titanium dioxide. Figure made in BioRender.com.

Nanomedicine, among its lines of research, stands out for the design of nanostructured systems capable of stabilizing drugs and releasing them following a controlled release, both with dosage and tissue selectivity [112]. These types of nanostructures are called nanocarriers, whose properties, such as large surface area and sub-micron sizes, allow the stabilization of higher loading or dosing per unit volume, as well as increasing the bioavailability of a drug where and when it is needed [113]. The nature of their surface allows manipulation of their surface chemistry to increase their biocompatibility and selectivity. In addition, there is great flexibility in the various routes of administration possible for these nanocarriers [114]. With respect to DA, as will be described below, several types of nanocarriers have been designed which satisfy the conditions set forth above for any DA delivery system. A summary on such nanotransporters is described in Table 1 and Figure 4.

#### 3.2.1. Polymers and Derivatives

Given their therapeutic potential and unique properties, biodegradable polymeric nanostructures have become an increasingly important approach for controlled DA delivery. To our knowledge, the first approach to DA loading in polymeric NPs was the work of Pillay et al. (2009) [115], who developed DA-loaded cellulose acetate phthalate NPs. The nanocarriers were physiochemically characterized and tested for controlled release in vitro and in vivo, providing favorable levels and controlled delivery of the neurotransmitter in rat cerebrospinal fluid over 30 days, with a peak at three days. In addition, the nanostructures were implanted in the frontal lobe parenchyma, a somewhat invasive route of administration, hence requiring the search for alternative nanostructured polymeric devices that would allow other routes.

In this sense, chitosan (CS), given its biodegradability, biocompatibility, bioactivity, nontoxicity, and polycationic nature, became one of the primary polymeric materials used for the design and synthesis of polymeric NPs [116]. There are many formulations for controlled drug release based on CS, including several for DA release. For instance, Trapani et al. (2011) [117] developed CS-based nanocarriers whose surface adsorbed DA. In vitro analysis of the nanostructures showed reduced cytotoxicity and a significant transport-enhancing effect compared to the control; in vivo experiments indicated that acute intraperitoneal administration of the polymeric NPs induced a dose-dependent increase in striatal DA output. More recent approaches have attempted to functionalize the CS structure by developing polymeric conjugates of esters and amides [118,119]. These improvements have allowed nanocarriers to transport DA across the BBB, preventing spontaneous autooxidation of DA and allowing for a nose-to-brain delivery. However, even though CS NPs exert neuroprotection by preventing DA oxidation and ROS generation, it has been observed that once released, DA generates increased H_2_O_2_ production. Interestingly, this is significantly lower than when pure DA is added. In this line, Ragusa et al. (2018) [120] observed an increase in the activities of superoxide dismutase and glutathione peroxidase enzymes, which could be related to the protective effect of CS NPs against DA-induced oxidative stress.

Another polymer that has received the most attention for the stabilization of other drugs and has been studied for the stabilization and release of DA is poly(D,L-lactic-*co*-glycolic acid) (PLGA) [121,122]. In this regard, Pahuja et al. (2015) [123] designed DA-loaded PLGA NPs for liberation in the SNpc and the striatum. The nanostructures allowed zero-order release up to 160 h after the onset of release, showing reduced clearance of DA from plasma, reduced formation of quinone adducts, and reduced DA autoxidation. When internalized in dopaminergic SH-SY5Y cells, the nanostructures caused no reduction in cell viability or morphological impairment. Similarly, intravenously administered NPs were able to cross the BBB in a rat model of 6-hydroxydopamine (6-OHDA)-induced PD, significantly reducing symptomatology without generating additional oxidative stress, degeneration of dopaminergic neurons, or structural modifications in the striatum and SNpc. To further improve the pharmacokinetic and pharmacodynamic profile of these DA-loaded PLGA NPs, Monge-Fuentes et al. (2021) [124] recently functionalized similar nanostructures with albumin, given its ability to permeate the BBB through receptor-mediated pathways. The resulting NPs significantly improved motor symptoms in vivo and regulated and restored motor coordination, balance, and sensorimotor performance in uninjured rats. Likewise, Tang et al. (2019) [125] developed PLGA-based NPs and co-modified them with borneol and lactoferrin for DA encapsulation. The low toxicity and increased cellular uptake of the NPs demonstrated that the functionalization improves drug transport to the brain via intranasal administration.

In addition to these polymers, DA has been stabilized in other types of polymeric NPs, albeit to a lesser extent. Rashed et al. (2014) [126] investigated the ability of DA-loaded polyvinylpyrbrolidone-acrylic acid (PVP/PAA) nanogel to deliver the neurotransmitter across the BBB. Recently, nanogels have emerged as promising drug delivery vehicles due to their ability to retain active molecules, macromolecules, and drugs, along with the capability to respond to external stimuli [127]. Along the same line, Ren et al. (2017) [128] developed injectable hydrogel nanocomposites capable of stabilizing a maximum of 2.0 wt% DA and releasing up to 8 mg of the neurotransmitter for up to 500 h; moreover, the hydrogel was cytocompatible. On the other hand, Trapani et al. (2021) [129] studied in vitro neurotransmitter release through its stabilization in oxidized alginate, a polysaccharide found in brown algae. Although the first approach required intraperitoneal administration, alginate-DA conjugates seemed to encourage further *ex vivo* and in vivo studies with a view to nose-to-brain administration. Finally, García-Prado et al. (2021) [130] loaded up to 60% DA into a polymeric nanostructure composed of 1,4-bis(imidazol-1-ylmethyl)benzene, which reduced apomorphine-induced rotations after nasal administration.

It is necessary, nonetheless, to highlight that, in many cases, the delivery mechanism of polymeric NPs involves degradation processes, which may result in the generation of toxic residual materials in the release medium [131,132]. Therefore, the design of new polymeric NPs for DA stabilization must consider the biocompatibility and stability of nanocarriers, particularly in post-release stages.

#### 3.2.2. Liposomes and Solid-Lipid NPs

Liposome-like nanostructures are a well-established type of drug carriers that have received significant attention due to their unique characteristics, such as enhanced drug delivery efficacy and biocompatibility [133]. Unlike polymeric NPs, liposomes are composed of phospholipids (which can come from nature or surfactants), giving them a biocompatibility far superior to other nanostructures [134]. In terms of controlled release of DA, liposomes offer the advantage of being able to maintain the physiological conditions necessary for its transport in its basal state (as in the case of presynaptic vesicles [135]), as well as the possibility of transporting it through the BBB [136]. During et al. (1992) [137] first achieved the encapsulation of DA in liposomes and its controlled release effect in vivo. The authors monitored DA levels in the striatal extracellular fluid by microdialysis and assessed apomorphine-induced asymmetric rotation for 25 days after stereotaxic implantation of the liposomes. Furthermore, they suggested the possibility of altering the membrane composition of the liposomes for encapsulation and release over extended periods of time. Subsequently, Jain et al. (1998) [138] managed to stabilize DA hydrochloride in positively charged liposomes for intraperitoneal administration, showing an improvement in the symptomatology of parkinsonian rats (with respect to L-DOPA administration), and proving the ability of the liposomes to transport DA across the BBB. In this same line of intraperitoneal administration, Zhigaltsev et al. (2001) [139] observed that increasing the DA/lipid ratio in DA-loaded liposomes employing an ammonium sulfate gradient results in complete compensation of dopaminergic deficiency in the rat brain. Having corroborated the possibility of DA encapsulation and transport in liposomes, research has focused on surface functionalization to improve encapsulation, recognition, and release in specific brain regions. For instance, to enhance DA stability, Trapani et al. (2018) [140] coated liposomes with thiolated CS, significantly protecting DA from autoxidation to a higher degree than previous CS NPs. Conversely, Khare et al. (2009) [141] developed glutamate-conjugated liposomes for receptor-mediated transcytosis DA delivery, whose in vivo administration results proved better over regular DA-liposome delivery. Similarly, Lopalco et al. (2018) [142] functionalized DA-loaded liposomes with transferrin, a hydrophilic carrier that regulates the extracellular iron level in human fluid and whose receptor has been targeted for improving the BBB transport of drugs [143]. Other surface functionalization approaches have included the use of the amyloid precursor protein [144] and virus glycoproteins [145], among others [146].

Even though phospholipid-based liposomes are nanosystems of great interest for controlled release, their low stability and high costs have recently led to the development of other emulsion-based NPs: solid lipid NPs (SLNPs). Constituted by a solid matrix that allows the controlled release of drugs, SLNPs combine the advantageous characteristics of NPs with those of lipid-based parenteral emulsions based on non-toxic and biodegradable lipid components [147]. Although widely used for drug delivery in neurodegenerative diseases in general [148], the use of SLNPs for DA stabilization is a fairly recent topic. Early physicochemical studies indicate the possibility of encapsulating DA with up to 81% effectiveness, as well as penetrating the BBB into the brain parenchyma [149,150]. Further research is currently ongoing to assess these nanocarriers’ in vivo performances for nose-to-brain delivery.

#### 3.2.3. Metal Oxide NPs

Metal oxide NPs, among polymeric NPs, liposomes, micelles, quantum dots, dendrimers, or fullerenes, are becoming increasingly important due to their potential use in novel medical therapies. Although in the design and synthesis of these nanostructures DA has served mainly as a surface functionalizing agent [151,152,153], considerable progress has been made in its stabilization through the main use of two types of metal oxide NPs: titanium(IV) dioxide (titania, TiO_2_) and silicon(IV) dioxide (silica, SiO_2_).

Titania has found its main application in nanomedicine due to its photoactivity, which has been used for photodynamic therapies [154]. Nonetheless, TiO_2_ NPs also offer significant advantages in drug delivery, enabling efficient pharmacokinetics and targeted delivery [155,156]. The first stabilization of DA in titania of which the authors are aware, was carried out by Vergara-Aragón et al. (2011) [157]. In this study, TiO_2_-DA complexes synthesized by the sol-gel method were implanted on the caudate nucleus of unilaterally lesioned rats, significantly recovering motor crossing and rearing behaviours. Subsequent studies identified that the oxidation process of DA was delayed for up to 30 days [158,159,160]; similarly, DA release from the device in vivo was corroborated up to 360 days post-implantation [161]. Other DA stabilization approaches on TiO_2_ have opted for the design of nanohybrid organic-inorganic composites, in which CS-DA composites were coated with titania [162]. The aforesaid allowed a containment of the neurotransmitter for 16 h, which could represent an alternative for the oral administration of DA. However, although promising for controlled delivery of DA, titania NPs have been associated with ROS production in brain microglia and dopaminergic neuron damage in vitro and in vivo [163,164,165]. Furthermore, TiO_2_ NPs have been observed to induce dose-dependent α-syn aggregation and fibrillation and impair the ubiquitin-proteasome system [166,167], making these nanostructures potential neurotoxic agents in the development of PD. Therefore, research on the use of nanostructured titania for DA delivery should evaluate their biocompatibility and nanotoxicity prior to further investigating their efficiency.

The alternative to titanium dioxide, which has received the most attention due to its controlled release properties and intrinsic biocompatibility, is silicon dioxide. Nevertheless, although widely used for the encapsulation and controlled release of drugs, genes, and others [168], to the authors’ knowledge, only one group has stabilized DA in SiO_2_ for controlled release. López et al. (2007) [169] developed a mesoporous nanosilica with a high specific surface area (500 m^2^/g) that entrapped DA and avoided its oxidation when synthesized in an inert atmosphere. When implanted in the striatum of hemiparkinsonian rats, the DA/SiO_2_ nanocarriers reversed the rotational asymmetry induced by apomorphine (up to 57%) with no signs of dyskinesias [170,171], an observation that is consistent with a slow and tonic DA release [172]. Although the results are promising, the mechanisms of DA release and its effect on the symptomatic amelioration need to be further elucidated.

Finally, it is important to mention recent oxide NPs used for the controlled release of DA: cobalt-ferrite (CF). De et al. (2021) [173] developed nanocomposites of CF with DA and polyethylene glycol (PEG), which allowed controlled drug release. However, the nanostructure was developed as an anticancer agent, its main mechanism being the induction of apoptosis and the generation of ROS, which makes these NPs unsuitable for potential treatments in PD.

#### 3.2.4. Inorganic NPs

In conjunction with organic controlled release devices, another group of nanostructures of great interest for drug delivery is inorganic NPs. Given their low toxicity and excellent properties, including wide availability, rich functionality, good biocompatibility, the potential capability of targeted delivery, and controlled release of carried drugs [174], inorganic NPs represent an excellent alternative for the stabilization and release of DA. In this regard, gold NPs (AuNPs) have generated promising results. Rout et al. (2020) [175] studied the binding interaction of DA with AuNPs using steady-state and time-resolved fluorescence spectroscopic tools. Similarly, Kalčec et al. (2022) [176] studied the DA loading efficiency for peptidoglycan monomer-coated AuNPs by fluorescence and UV-Vis measurements. The results of both groups suggest the possibility of using these nanostructures as drug delivery models in DA-related diseases, whose functionalization could probably fit multiple functions, i.e., efficient drug-loading, BBB-penetrating, and CNS-targeting system DA. However, in vitro and in vivo investigations should further confirm these assumptions.

Another type of inorganic nanostructures capable of DA release are those called quantum dots (QDs), semiconducting NPs with unique size and shape-dependent optoelectronic properties [177]. In this respect, Malvindi et al. (2011) [178] developed highly fluorescent cadmium selenide/cadmium sulfide QDs whose functionalization with PEG allowed for controlled DA release by enzymatic degradation with esterases from the porcine liver. Subsequently, Khan et al. (2015) [179] anchored DA to water-soluble carbon QDs, which proved biocompatibility under physiological conditions in vitro and to be non-toxic in vivo, as they did not inflict any anatomical distortions or negative effects on tissues. Furthermore, Mathew et al. (2020) [180] conjugated similar carbon QDs with CS, which demonstrated around 97% cell viability, DA encapsulation efficiency of >80%, and the sustained release of DA.

Finally, the last reported approach for stabilizing DA in inorganic nanostructures is the work of Mathew et al. (2021) [181], who developed copper sulfide composites with CS. The main difference between these NPs concerning others lies in their release mechanism, which involves irradiation with infrared light. The dissipated heat causes the release of the encapsulated DA, hence making this photo-controlled technique a potential method to control targeted release in specific areas of the brain, such as the SNpc or the striatum, in the case of PD.

**Table 1:** Nanocarriers tested for controlled DA delivery.

Type of Nanostructure	Nanocarrier + Functionalizing Agent	Administration Pathway	Ref.
Polymeric	Cellulose acetate phthalate	Stereotaxic surgery	[115]
	Chitosan	Intraperitoneal	[117]
	Chitosan + esters/amides	Intranasal	[118,119,120]
	Poly(lactic-co-glycolic acid)	Intravenous	[123]
	Poly(lactic-co-glycolic acid) + albumin		[124]
	Poly(lactic-co-glycolic acid) + borneol/lactoferrin	Intranasal	[125]
	Polyvinylpyrrolidone/Polyacrylic acid nanogel	Intraperitoneal	[126]
	Hydrogel	‡	[128]
	Oxidized alginate	Intraperitoneal	[129]
	1,4-bis(imidazole-1-ylmethyl) benzene	Intranasal	[130]
Lipidic	Liposome	Stereotaxic surgery	[137]
	Liposome + stearylamine	Intraperitoneal	[138]
	Liposome	Intraperitoneal	[139]
	Liposome + thiolated chitosan	‡	[140]
	Liposome + glutamate	Intraperitoneal	[141]
	Liposome + transferrin	‡	[142]
	Liposome + amyloid precursor protein	Intraperitoneal	[144]
	Liposome + virus glycoproteins	Intravenous	[145]
	Solid lipids	‡	[149,150]
Metal oxide	Titanium dioxide	Stereotaxic surgery	[157]
	Titanium dioxide + chitosan	Oral	[162]
	Silicon dioxide	Stereotaxic surgery	[169]
Inorganic	Gold NPs	‡	[175,176]
	Selenide/cadmium quantum dots + Polyethylene glycol	‡	[178]
	Carbon quantum dots	Intravenous	[179]
	Carbon quantum dots + chitosan	‡	[180]
	Copper sulfide + chitosan	‡	[181]

‡ Only tested in vitro.

## 4. Oxidative Stress Reduction, Protein Aggregation Inhibition, and Selective Proteolysis on Parkinson’s Disease

### 4.1. Nanozymes as A Potential Treatment of Parkinson’s Disease

Although essential in treating the symptomatology, the restitution of basal DA levels in PD patients does not impede the progression of the disease. Indeed, in advanced stages, dopaminergic neuronal death can become aggravated to levels that render dopaminergic restitution useless. Therefore, the key to stopping this neurodegenerative disease lies in treating the underlying causes that lead to neuronal death and, thus, dopaminergic depletion. From the physiological perspective followed in this work, the treatment of the pathophysiological processes responsible for the progression of the disease could be achieved by artificially optimizing the normal homeostasis preservation processes described above for healthy dopaminergic neurons. This can be achieved by designing nanostructures capable of replicating biological catalysis.

The dawn of such structures dates back to the discovery of the first nanomaterials capable of enzyme-like activity in the 1990s, which received the name of nanozymes by Scrimin and co-workers [182]. In 2007, the inherent peroxidase-like activity of Fe_3_O_4_ NPs was verified [183]. Since then, interest in nanozymes has increased across a variety of sectors, and several nanomaterials with properties like those of enzymes have been suggested, including metals (gold, silver, platinum, palladium, and others), metal compounds (magnetite, cerium(IV) oxide, manganese(IV) oxide, copper sulfide, manganese selenide), non-metals (carbon dots, fullerenes), and non-metal compounds (graphitic carbon nitrides, graphene oxide) [184]. When compared to natural enzymes, nanozymes offer several beneficial properties, such as higher catalytic stability, lower production costs, higher tolerance to surrounding environments (can work in a broader range of pH and temperature), large surface areas (which can be easily modified), good stability, tuneable activity, specific environment responsiveness, adjustable biological functionalization (which can optimize internalization, circulation, and absorption in tissues or cells), and intrinsic physicochemical properties (magnetism, photothermal qualities, and others) that allow for remote control (through magnetic fields, lasers, and ultrasounds, among others) [184].

**Figure 5:**
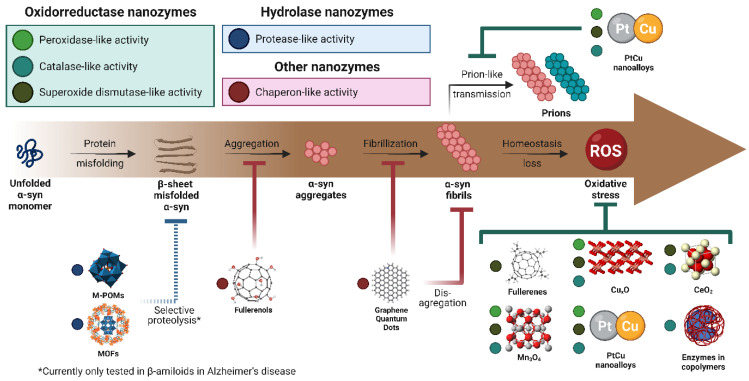
Current research on nanozymes for oxidative stress reduction, protein aggregation inhibition, and selective proteolysis of misfolded alpha-synuclein. Nanozyme research and their application in the different stages of the pathological loss of proteostasis observed in Parkinson’s disease. Enzyme-like activities are depicted in different colors, and nanozymes with oxidoreductase-, hydrolase, and other-like activities are presented inhibiting their target. * M-POMs and MOFs selective protease-like activities have only been observed for β-amyloids in Alzheimer’s disease. ROS: reactive oxygen species; α-syn: alpha-synuclein; M-POMs: metal-substituted polyoxometalates; MOFs: metal-organic frameworks; Pt: platinum; Cu: copper; Cu_x_O: copper oxide; CeO_2_: cerium dioxide; Mn_2_O_4_: manganese oxide. Figure made in BioRender.com.

Current nanozyme enzymatic-like activities are mainly divided into oxidoreductases (oxidases, peroxidases, catalases, superoxide dismutases, and nitrate reductases) and hydrolases (nucleases, esterases, phosphatases, silicateins, and proteases) [185]. Each suggests possible applications in oxidative stress reduction, inhibition of protein aggregation, and selective proteolysis of misfolded proteins. Therefore, the following sections will describe the most recent proposals based on nanozymes for the potential treatment of PD. The current advances in this research are summarized in Figure 5 at the end of the section. For a more detailed description of other types nanozymes, refer to Yang et al. (2021) [184] and Wu et al. (2019) [186].

### 4.2. Nanozymes for Oxidative Stress Reduction

With oxidative stress being one of the leading causes of the process of degeneration and cell death in dopaminergic neurons during PD, as well as a precursor of α-syn misfolding and aggregation, the oxidoreductase-like activity of some nanozymes, especially peroxidase, catalase, and superoxide dismutase (SOD)-like activities, could represent a relief to this damage. In fact, several ROS-scavenging nanozymes have been developed and shown significant promise for tissue repair and regeneration [187]. Therefore, for the purposes of this paper, we will limit ourselves to reviewing those nanozymes that have been tested in models of neurodegenerative diseases, particularly in PD.

#### 4.2.1. Fullerene-Based Antioxidant Nanozymes

The first ROS-scavenging nanozyme for PD the authors are aware was developed by Dugan et al. (2014) [188]. They administered C3-modified C60 fullerenes with SOD-like activity into a nonhuman primate PD model and observed improved movement scores and increased striatal DA levels. Moreover, fullerene optimization with C3 improved the aqueous solubility of the lipophilic core, which provided an enhancement in blood–brain barrier (BBB) penetration.

#### 4.2.2. Metal-Oxide-Based Antioxidant Nanozymes

However, more recent research has turned to metal oxides as antioxidant nanozymes. Singh et al. (2018) [189] were the first to test manganese oxide (Mn_3_O_4_) nanozymes with SOD, catalase, and glutathione peroxidase activity in vivo. The high Mn^2+^/Mn^3+^ ratio, large specific surface area, and pore size endowed them with such antioxidant effectiveness and stability to scavenge intracellular and mitochondrial ROS. The above inhibited the microglial activation and lipid peroxidation while protected the TH in the striatum of PD model mice. Independently, Xu et al. (2021) [190] developed similar Mn_3_O_4_ nanozymes and observed that cellular uptake was mediated by caveolin, with biodegradation being reached at 60 days.

On the other hand, the SOD and catalase-like activity of cerium dioxide (CeO_2_) has been evaluated by several groups. For instance, Mook-Jung et al. (2016) [191] described CeO_2_ nanozymes functionalized with lipophilic cation triphenylphosphonium, which endowed them with selectivity for Alzheimer’s disease mouse model mitochondria. In this study, the NPs mitigated reactive gliosis and morphological mitochondria damage observed in these mice. In the same line, Hyeon and co-workers transferred the study to a mouse PD model, testing three types of CeO_2_ nanozymes [192]. By boosting SOD and catalase enzyme activities, the authors inhibited the activation of microglia cells and lipid peroxidation by removing ROS inside and outside the cells while protecting the TH.

Another type of metal oxide nanostructure tested for its oxidoreductase-like properties in the field of PD treatment is copper oxide (Cu_x_O). Developed by Hao et al. (2019) [193], the Cu_x_O nanozymes mimicked the activities of peroxidase, SOD, catalase, and glutathione peroxidase and eliminated ROS in a cellular model of PD. Furthermore, their application also rescued the memory loss of the PD mice.

#### 4.2.3. Metal-Based Antioxidant Nanozymes

Recently, Liu et al. (2021) [194] evaluated the antioxidant effect of a platinum-copper (PtCu) nanoalloy in an in vivo model of sporadic PD by intrastriatal injection of α-syn preformed fibrils (PFFs), which has been used extensively to study the transmission of misfolded α-syn aggregates through the brain [195]. Surprisingly, the peroxidase, catalase, and SOD-like activity of the PtCu nanozymes not only enabled them to scavenge ROS present in cell cultures, but also inhibited the prion-like cell-to-cell transmission of α-syn PFFs both in vitro and in vivo. According to the authors, further work will seek to evaluate the impact of these nanozymes on the symptomatology associated with the synucleinopathy observed in PD. Taken together, these data provide a concept of proof that this redox metal–metal nanozyme can be considered to be developed as a therapeutic strategy against pathologic α-syn spreading in PD and related a-synucleinopathies. In the same line, selenium nanozymes functionalized with glycine were observed to prevent brain oxidative stress and neurobehavioral abnormalities when administered in parkinsonian rats [196].

#### 4.2.4. Polymer-Enzyme-Based Antioxidant Nanozymes

Finally, the other significant approach in the design of nanostructures with antioxidant properties involves the development of catalases stabilized in nanostructured copolymers and embedded in macrophages for their controlled release [197]. This work from the Batrakova and collaborators group has generated a facilitated transport mechanism that allows the passage of nanoformulated catalases across the BBB into endothelial, neuronal, and glial cells [198]. In in vivo models of PD, these nanozymes have been able to reduce microgliosis and double DA production in dopaminergic neurons due to their antioxidant effect [199].

### 4.3. Nanozymes for Protein Aggregation Inhibition

Abundant evidence has highlighted the aggregation of α-syn into insoluble amyloid fibrils as the key cause in the progression of genetic and familial PD [200]. For this reason, considerable effort has been focused on identifying strategies that inhibit this fibrilization of α-syn [201]. Interestingly, although there are several studies concerning the interaction of nanostructures with α-syn and their impact on α-syn aggregation [202,203], our literature search showed that only two nanozymes have been synthesized with chaperone-like activity to inhibit misfolded α-syn aggregation.

The first is graphene quantum dots developed by Kim et al. (2018) [204,205]. These nanostructures were able to inhibit fibrillation of monomeric misfolded α-syn, as well as interact with mature fibrils by activating their disaggregation. Furthermore, the authors observed that these quantum dots reduced the formation of LBs and neurites and prevented cell-to-cell transmission of pathological α-syn. In their in vivo models, the quantum dots crossed the BBB. Collectively, these results showed that graphene quantum dots are the optimal therapeutic candidate for anti-PD and related α-synucleinopathies therapy, with no appreciable in vitro and long-term in vivo toxicity.

The other nanostructures with catalytic chaperone-like properties are the hydroxyfullerene derivatives (fullerenols) developed by Sun et al. (2019) [206], who investigated their effect on α-syn amyloidogenic center aggregation. Interestingly, unlike normal hydrophobic fullerenes, fullerenols were able to inhibit aggregation since hydrogen bonding between hydroxyls and peptide backbones interrupted the formation of β-sheets between peptides adsorbed onto the surfaces of fullerenols or fullerenol nano-assemblies due to hydrophobic interactions. Thus, both cross-β aggregates and β-barrel intermediates were significantly suppressed.

Thus, having corroborated the possibility of inhibiting the aggregation of α-syn misfolded and dismantling fibrils already formed, further research is needed on these and new nanostructured materials that exert this chaperone-like activity.

### 4.4. Nanozymes for Selective Proteolysis

Developing effective heterogeneous catalysts for the controlled transformation of large and complex biomolecules, such as proteins, has been a particular challenging for nanozyme researchers. Despite their explosive growth in the last decade [186], most enzyme-like nanostructures exhibit redox-type activities, such as peroxidase, oxidase, superoxidase, and catalase [207]. Hydrolysis reactions, on the other hand, have been little explored, with phosphodiesterase-type nanozymes mainly being reported [208,209]. However, although few, proteolytic nanostructures have been developed recently. They are based on metal-oxo clusters, a vast class of compounds regarded as discrete soluble intermediates of polymeric metal oxides that possess physicochemical properties extremely attractive for the development of catalysts [210]. In this section of the review, we will limit ourselves to briefly describing the main findings. For a complete description, refer to Azambuja et al. (2021) [211].

#### 4.4.1. Polyoxometalate (POM)-Based Nanozymes

The first artificial proteases developed were anionic polyoxometalates (POMs), metal-oxygen clusters rich in structural and configurational diversity and nuclearity, electronic and magnetic properties, acid or basic nature, and widely varying charge density [212]. Parac-Vogt and co-workers improved their catalytic properties by the incorporation of strong Lewis acidic metals [211], such as Zr(IV), Hf(IV), and Ce(IV) [213,214,215]. These metal-substituted POMs (M-POMs) have been capable of selectively hydrolyzing a wide diversity of proteins under physiological pH conditions, including human serum albumin, transferrin, hemoglobin, ovalbumin, myoglobin, lysozyme, and cytochrome c, among others [211], cleaving them into discrete fragments within 24–48 h [216]. Mechanistic experiments and density functional theory calculations have confirmed that the metal, through free coordination sites to the carbonyl oxygen atom of the peptide backbone, accelerates peptide bond cleavage through a Lewis acid activation mechanism [217]. In addition, the POM framework is responsible for engagement with specific regions of the protein (especially, the positively charged regions [218]) in a non-covalent enzyme-like interaction [219], placing the embedded metal close to the position where it will not only selectively cleave the bond, but also impart relaxation to the protein secondary structure upon binding, which could be essential for making potential cleavage sites more accessible to the embedded Lewis acid [220].

In the field of misfolded proteins and neurodegenerative diseases, the group of Gao and co-workers was the first to develop M-POMs capable of depleting beta-amyloid (βA) aggregates through nanoproteolysis [221]. Previously, Gao et al. (2014) [222] had synthesized a POM capable of inhibiting Aβ aggregation in vitro by selective binding. Knowing that native serine proteases act through the catalytic triad Serine (nucleophilic attack)–Histidine (electron transfer)–Aspartate (electron donor) [223], the intrinsic electronegativity of POMs (similar to that of the hydroxyl group of Serine in native serine proteases) was optimized by incorporating a Histidine-rich heptapeptide for electron transfer. Finally, to further improve electron transfer, they stabilized AuNPs, which not only made the synthesis more efficient by serving as a scaffold for the POM-peptide composite, but also endowed the nanocomposite with the ability to cross the BBB. Similarly, they have also developed a hybrid of cerium oxide (CeO) and a POM with both proteolytic activity towards βA aggregates (from POM) and superoxide dismutase activity to reduce intracellular ROS (from CeO) [224]. The authors reported a proteolytic enzyme performance of 64.71 U/mg and assumed that surface charge played an important role in the hydrolytic reactions. In this regard, although focused on the selective proteolysis of βA aggregates, the work of Gao and co-workers, together with that of Parac-Vogt and co-workers, sets a milestone for the development of POM nanozymes capable of selectively degrading misfolded proteins. The key to transferring this proteolytic activity to other misfolded proteins (such as α-syn) would lie in properly functionalizing their surface to make them selective towards the target protein.

#### 4.4.2. Metal-Organic Framework-Based Nanozymes

Along with the development of POMs, another type of crystalline porous material known as metal-organic frameworks (MOFs) began to be studied. These are structures formed by metal ions coordinated to organic ligands, which have found application in gas storage and catalysis due to their vast surface areas [225,226,227], as well as adsorbents for peptide enrichment, protein conjugation, or enzyme immobilization, although the latter are still limited [228]. Furthermore, although rarely, MOFs have also shown enzyme-like properties, mainly as peroxidase mimics [229,230]. Nonetheless, while unknown for a long, the intrinsic protease-mimic potential of MOFs has started to be investigated. In fact, the research group of Parac-Vogt has begun to dabble in the development of MOFs with peptidase-like activity based on Zr_6_O_8_ clusters such as MOF-808 [231], UiO-66 [232], and NU-1000 [233]. Notably, all three MOFs hydrolyzed the Gly-Gly dipeptide faster than their Zr-POMs counterparts. Similarly, Li et al. (2014) [234] synthesized a MOF in which copper(II) was stabilized; its octahedral structure allowed hydrolysis of albumin and recovery of the nanostructure for reuse up to ten times.

Thus, with POMs, MOFs could also represent another possible group of nanostructures capable of hydrolyzing misfolded proteins once their selectivity has been further explored by functionalizing their surface with protein recognition agents.

## 5. Perspectives on Nanomedicine-Based Therapies for Parkinson’s Disease

As our knowledge of the pathophysiology of Parkinson’s disease deepens, new approaches derived from nanomedicine begin to represent alternatives for the inhibition of the associated symptomatology, primarily through the restitution of physiological levels of depleted DA and of the underlying causes of neuronal death that determines the progression of SNpc neurodegeneration, as is the case of α-syn misfolding and aggregation. Up to this point, this work has focused on presenting the most recent developments in controlled DA release, reduction of oxidative stress, and aggregation of misfolded proteins. However, although significant, their clinical translation requires further research on certain parameters related to the optimization of their administration, bioavailability, biocompatibility, and selectivity to reduce the possibility of side effects due to unanticipated nanotoxicity. Therefore, this section will describe the requirements that any research must satisfy to obtain nanomedicine-based therapies for PD.

### 5.1. Non-Invasive Administration Methods for NPs

#### 5.1.1. Overcoming the Blood–Brain Barrier

Diseases of the central nervous system (CNS) require the transport of drugs and therapeutic compounds into the brain, in the regions directly related to the disease. However, the impermeable nature of the BBB represents a limitation when designing new treatments, especially for hydrophilic and sizeable molecular weight structures [235]. The above is associated with downfalls of dose-limiting systemic side effects and tough dosage regimes [236]. In this sense, the traditional approach has opted for stereotactic injections which allow the implantation of drugs and other structures directly in specific regions of the brain with a high precision [237]; however, the invasive nature of surgery makes its use impossible as a method of administration in nanotherapies, especially when their application must be recurrent. Therefore, the key to developing new non-invasive delivery devices depends on further investigating the behavior of the BBB to allow the passage of compounds from the primary means of administration: intravenous, intramuscular, subcutaneous, and inhaled, among others.

BBB passage limitations are due to the presence of tight junctions between the cells and their high resistance (1500–2000 Ω • cm^2^) due to the encapsulation of capillaries by pericytes and astrocytes. [238,239]. Only small molecules (water, gases, lipophilic compounds) can pass through passive transcellular diffusion, while other molecules with hefty electrical charges, polarity, and hydrophilicity require specific proteins for active transport pathways [240]. An initial approach, in this sense, has consisted in the temporary disruption of tight junctions, which has been achieved by osmotic pressure [241], microbubbles [242], and ultrasound [243]. Though, the process damages the integrity of the BBB causing an uncontrolled flow of drugs, toxins, or molecules into the CNS during the opening of the tight junctions, making these methods highly risky [244].

In this regard, since active transport requires for specific interactions between molecules and the BBB, the new proposals have opted for the surface functionalization of the compounds to be delivered into the brain. For nanostructures, coatings have included peptides [245,246], proteins [247,248], nucleic acids [248,249], and antibodies [250,251]. This functionalization endow the NPs with physicochemical properties that facilitate BBB endothelial cellular uptake through passive diffusion, carrier-mediated transport, receptor-mediated endocytosis, and absorption-mediated endocytosis [252]. Albumin, for example, has been widely used to facilitate the passive transport of nanostructures with up to 8 times higher efficiency than non-functionalized NPs [253]. Similarly, conjugation with glucose or glucose analogs has enabled passive diffusion via glucose transporters widely expressed in endothelial cells [254]. However, among pathways, the receptor-mediated transcytosis (RMT)-based transport system has been identified as the most efficient and promising strategy for BBB permeabilization [255]. Because the RMT process is tightly controlled, RMT-initiated nanopreparations provide an ideal platform for selective delivery to the CNS as compared to passive transport systems [256]. The receptors expressed on the luminal side of brain endothelial cells include transferrin receptors, scavenger receptors, insulin receptors, and lipoprotein receptors [257]. Hence, transferrin-related ligands (peptides, proteins, antibodies) have been tremendously explored in terms of their ability to promote the RMT across the BBB from systemic administration [258,259]. Indeed, in the field of nanomedicine-based therapy research for PD, Huang et al. (2010) [260] studied a gene therapy using lactoferrin-modified NPs in a chronic rotenone-induced PD model. Similarly, Yan et al. (2018) [261] observed excellent biodistribution in the rat brain for lactoferrin-modified NPs.

Thus, the development of anti-PD nanostructures, whether for controlled release or with intrinsic therapeutic properties, must focus on optimizing the coating components to enhance transport through the BBB and, thus, enable the potential development of systemic delivery pathways for the new compounds.

#### 5.1.2. Nose-to-Brain Administration of NPs

Nonetheless, in addition to surpassing transportation across the BBB, systemic drug administration deals with further problematic scenarios. Oral administration, for instance, exhibits poor intestinal absorption, slow onset of action, and extensive gut and hepatic-first pass metabolism; similarly, injectable drugs cope with injection site-reactions, self-administration compliance issues, and needle phobia [262]. It has been observed, as an alternative, that the intranasal administration of drugs avoids most of the discomforts associated with other routes [263], offering an unprecedented opportunity to target the brain. This route improves into-brain drug administration due to the presence of permeable epithelial tissue and the extensive vascularization of the mucosa and laminae, allowing for an increased drug availability in the brain, avoiding first-pass metabolism, providing comparable bioavailability to the intravenous route, and reducing systemic side effects [264].

However, despite the various advantages associated with the intranasal route, the administration of traditional therapeutic agents remains problematic due to the constraints imposed by the geometry of the nasal cavity, namely the limited volume of drug delivery and the limited surface area of the olfactory region [265], and the physiological process, such as short residence times and regular mucociliary clearance [266]. In view of this situation, new nanomedicine approaches have sought to optimize nasal permeability, adhesion to the mucosa, and homogeneity in absorption through the surface functionalization of nanostructures with natural (chitosan, gelatin, lectins, gum, and alginate), semi-synthetic (celluloses), and synthetic (polyacrylates and thiomers) mucoadhesive agents [267], thereby improving systemic bioavailability and reducing the variability of nasal absorption [268]. Indeed, as described in the section “Nanocarriers for controlled release of dopamine”, there are currently several nanostructures capable of stabilizing DA in its basal state whose surface engineering has endowed them to be delivered nasally and cross the BBB en route to the brain, unlike their non-functionalized counterparts. [268]. In conclusion, these functionalized nanocarriers offer more potent drug binding to the mucosal surface, which increases drug concentrations at the site of action. Their enhanced loading, controlled release, absorption and adhesion, increased stability, biocompatibility, and biodegradability make them ideal for drug delivery to the brain [269].

Nevertheless, although promising, nasal nanoformulations (both for controlled drug delivery and enzyme-like activity) still require much more research prior to becoming viable treatments. Despite the advantages of current functionalized nanocarriers, the high and frequent dose of the formulation still represents a limitation due to irritation of the nasal mucosa [270]. In addition, the protective barriers of the nasal cavity restrict the efficacy of intranasal therapy, as currently, only ≤1% of the compound reaches the brain after intranasal administration [270]. This problem requires more efficient nanostructured excipients since the alternative of increasing formulation quantities is impossible due to the small quantities (100–200 μL) per dose allowed by the nasal cavity, given its relatively low volume (25 cm^3^) [271]. In addition, any nasal nanoformulation must produce no aggressive odors, as well as be inert upon the conditions of tonicity, viscosity, and pH (5.0–6.5) present in the mucosa [272]. Furthermore, contradictorily, current conflicting results in the research of intranasal administration of NPs in terms of discrepancies in absorption rates and concentrations corroborate the crucial need for further research to address the abovementioned points [273].

### 5.2. Improved Selectivity for NPs

Although paramount in the development of nanotherapies for PD and other CNS diseases, efficient access to the brain is only the first of the challenges when designing optimized nanostructures. Once in the brain tissues, the NPs must be directed by chemotaxis towards the therapeutic targets and carry out their mechanisms of action only in the presence of the affected tissues since a deregulated action may result in a diminished therapeutic effect or side effects associated with alterations of other physiological processes. For example, in the case of DA nanocarriers, dysregulated release in areas other than the SNpc—say, the striatum—could lead to dysregulation of motor activity or oxidative stress, as an increase in extracellular dopamine can both overstimulate dopaminergic receptors on striatal neurons and oxidize and generate detrimental ROS. Similarly, non-selective proteolytic nanozymes, for instance, could degrade vital proteins and destabilize the homeostasis of the cell. In this regard, development of anti-PD nanostructures must ensure selectivity in their action mechanisms.

#### 5.2.1. Surface Functionalization for Optimized Selectivity

Selectivity towards disease-specific structures is especially critical for oxidoreductase-like and proteolytic nanozymes since a lack of substrate specificity could lead to uncontrolled molecule destabilizations. This problem has been partly solved by combining enzymes and nanozymes into hybrid structures. Nonetheless, this approach reduces the stability and increases the production costs of the catalytic system as a whole [274]. Therefore, following the physiological perspective, the incorporation of recognition pockets (as in the case of natural enzymes) is under research as a promising alternative.

Indeed, several systems have been used to coat the surface of NPs for selective protein identification through tailored capping and molecular imprinting. For the former, successful approaches have opted for recognition structures such as aptamers, receptors, and ligands. For instance, Hsu et al. (2016) [275] achieved a tunable peroxidase-like activity through aptamer-modified AuNPs with high selectivity (>1000-fold over other proteins) and sensitivity (detection limit ~0.5 nM). Similarly, Zhang et al. (2020) [276] fabricated Fe_3_O_4_ nanostructures with a peroxidase-like activity whose functionalization with an aptamer selective to platelet-derived growth factor BB achieved detection levels of up to 10 fM. In the field of ligands, You et al. (2008) [277] functionalized AuNPs with leucine and phenylalanine residues to target alpha-chymotrypsin and cytochrome c surfaces. The researchers observed that the binding affinity increased as new hydrophobic sites were incorporated into the NPs. Similarly, Bizzarri et al. (2007) [278] made use of a bifunctional molecule with a thiol group at one end to bind to AuNPs, and with a diazonium moiety capable of reacting with electron-rich aromatic side chains of proteins at the other. In doing so, they could detect thrombin at up to 0.5 pM. The authors indicated that the method could be easily implemented in a multiplexing approach by preparing capture substrates with the different recognition elements organized in arrays. Notably, the use of surface-functionalization with ligands has already yielded results in new potential nanotherapies for PD. Indeed, You et al. (2018) [279] incorporated rabies virus glycoprotein on the surface of polymeric NPs loaded with DA and deferoxamine, which significantly increased their selectivity for acetylcholine receptors of dopaminergic neurons, allowing their internalization and drug release inside these cells. Similarly, to further target these neurons, neurotransmitters binding to the dopaminergic D2 and D3 receptors have been proposed as useful ligands for targeting PD brains [280].

The other field of protein recognition in NPs is based on molecular imprinting. The process generates specific recognition sites through the polymerization of functional and crosslinking monomers in the presence of the target protein molecule, which acts as a template of the molecule of interest [281]. One of such monomers is DA itself, whose polymerized form (polydopamine) mimics adhesive proteins [282]. Xia et al. (2013) [283], for example, were able to separate bovine hemoglobin from cattle whole blood using SiO_2_ NPs imprinted with polydopamine. Subsequently, Han et al. (2020) [284] further optimized the imprinted polydopamine coating in similar SiO_2_ NPs with a slightly crosslinked nonlinear PEG layer, reducing nonspecific adsorption and increasing the imprinting factor from 2.6 to 6.4, which translates into a significant enhancement of both recognition selectivity and specific binding capacity to the imprinted NPs. Moreover, monomers with opposite charge to the substrate have also been used to create binding pockets not only complementary in shape to substrate molecules, but also electrostatically attractive [285]. Finally, the most recent studies have opted for molecularly imprinting polymeric NPs with aptamers [286]. In this sense, aptamers act not only as ligands, but as the polymeric structure responsible for creating the recognition pockets in the surface of the NPs. For instance, Shoghi et al. (2018) [287] coated magnetic NPs with an imprinted polymer made of aptamers, achieving >97% effectiveness in albumin recognition.

#### 5.2.2. Towards a Specific Enzyme-Like Activity for Misfolded Alpha-Synuclein

Developments in recognition and selective activity by specific proteins of NPs suggest the possibility of functionalizing nanozymes with oxidoreductase, chaperone, and proteolytic properties to act only in the presence of pathological forms of α-syn. Indeed, recent studies have successfully detected different conformations of α-syn by functionalized NPs. Sun et al. (2017) [288], being the first, reported the detection of soluble oligomers of α-syn using aptasensors (DNA aptamers) incorporated into AuNPs. You et al. (2019) [289], in turn, improved their sensitivity to 10 pM by coating the AuNPs with optimized α-syn specific aptamers. Furthermore, Guo et al. (2020) [290] designed a nanohybrid cobalt-manganese-zeolitic imidazolate framework nanosheets and carbon nanofibers-based electrochemical aptasensor with a limit of detection of 52.5 fM.

Aptamers are not the only surface functionalizing agents used for α-syn detection. Coating with anti-α-syn antibodies has enabled the development of nanostructured immunosensors with high sensitivity toward α-syn. For example, An et al. (2010) [291] prepared TiO_2_ nanotubes optimized with AuNPs and functionalized with primary antibodies, reporting a detection limit of 34 pg/mL. Similarly, bioconjugates of AuNPs coated with dendrimers and anti-α-syn antibodies have been fabricated, which could detect up to 0.135 pg/mL α-syn in cerebrospinal fluid [292]. On the other hand, regarding the molecular imprinting technology, Ma et al. (2020) [293] molecularly imprinted two-dimensional graphene nanosheets for direct detection of α-syn. The prepared sensor showed higher selectivity towards the analyte molecule, with a limit of detection of 3.5 × 10^−5^ ng/mL. A more detailed summary of reported nanosensors for α-syn measurement is offered by Chauhan et al. (2021) [294].

Thus, following the logic described above regarding the optimization of specificity as a function of surface functionalization, the use of aptamers, antibodies, and coatings by molecular imprinting specific for the pathological forms of α-syn could allow the design of highly selective nanozymes, especially those designed to carry out protease-like activity on the misfolded monomers of the protein. In fact, as mentioned for M-POMs, this principle has already been used to design proteolytic nanostructures capable of selectively degrading βA aggregates. Therefore, more research must be carried out to further identify all the potential pathological forms of α-syn, develop ligands and coatings that are highly affine to these proteins, and, hence, design selective nanopreparations capable of catalytically degrading misfolded α-syn monomers and aggregates, without affecting regular α-syn or any other protein.

### 5.3. Limitations and Safety Considerations for Nanomedicines in Parkinson’s Disease

In spite of the above, it is necessary to emphasize that research on nanomedicine for PD must not only focus on efficiency, but also consider that any nanomedical proposal requires specific studies on nanotoxicology. Only by knowing and predicting NPs’ quality, safety, and selectivity through the collective knowledge of neurobiochemistry, neurophysiology, and pharmaceutics, can their applications and reserves be defined and, hence, their clinical translation [295]. Below, we briefly discuss the known and potential nanotoxic effects on neural cells and tissues, emphasizing neural pathology considerations.

#### 5.3.1. The Limits of Surface Modification in NPs

Although surface optimization through incorporation of functionalization agents seems to be the proper strategy to improve transport through the BBB, allow for non-invasive administration routes, and enhance selectivity, there is a limit. Indeed, the excessive modification of NPs surfaces with immoderate amounts of targeting ligands can reduce selectivity by increasing binding to both target and non-target cells [296]. It is therefore important to find a balance between targeting efficiency and improving selectivity. In this regard, the group of Pun and coworkers developed a method to precisely control the density, folding direction, and folding structure of ligands in the final polymer structure. As an example, they found that the optimal density of Tet1 ligand in the in vivo model was around 3–5% (mol% by feed) to ensure specific selectivity in neuronal cells [297].

#### 5.3.2. Nano-Derived Oxidative Stress

When developing NPs with catalytic properties (and, even, those intended only for drug delivery), it must be ensured that their activity does not lead to the generation of oxidative stress complementary to that present in PD. Among the structures to what out for, titanium dioxide, gold, and transition metal NPs stand out. The former has been shown to accumulate in brain tissues (mainly the hippocampus) for up to 4 months after oral exposure [298,299], inducing mitochondrial dysfunction at 2 h and a significant increase in the activity of peroxidases, catalases, and dismutases [300]. On the other hand, although their biosafety in short-term treatments is accepted, the accumulation and lack of biodegradability of AuNPs generate concern, especially due to lipid peroxidation and DNA damage by oxidative stress that generate [301]. Finally, in general, transition metals (mainly iron, manganese, gold, and copper) are known to trigger the formation of oxygen free radicals and, thus, oxygen toxicity through the Haber-Weiss-Fenton reaction [302]. The above increases neurodegenerative damage, so the use of metal NPs in anti-PD treatments should be considered with special attention, especially in therapies requiring extended periods of time.

#### 5.3.3. Nano-Derived Autophagy and Lysosomal Dysfunction

Apart from ROS production, it must be considered that nanomaterials are often associated with lysosomal dysfunction through lysosome membrane permeabilization [303]. This phenomenon may result from nonbiodegradable particle sequestration into lysosomal compartments, such as NPs derived from fullerenes and certain dendrimers [304]. Similarly, NPs with positive surface potentials can cause excessive activity of proton pumps in lysosomes and, with that, osmotic swelling and rupture [305]. Furthermore, nanomaterials can also form adducts with cytoskeletal proteins, which can disrupt vesicle trafficking, avoiding the fusion between autophagosomes and lysosomes [306,307], thus altering autophagy, as well.

### 5.4. Towards Clinical Translation

In summary, uncontrolled activity in NPs may not only alter the physiological processes of neurons but also cause further neurodegenerative damage rather than provide a cure. Therefore, as progress continues in the development of new anti-PD nanomedicines, the following issues need to be concomitantly resolved prior to any clinical translation: (i) what the consequences of nanomaterial interaction with PD pathological states are?; (ii) is brain homeostasis altered after long periods of nanomaterial administration?; (iii) is it possible to inhibit adverse effects by optimizing the surface composition of the nanomaterials?; and (iv) does the cost/benefit ratio justify the use of the proposed nanomedical therapy? In this regard, only after a thorough recognition of any potential neurotoxicity arising from the application of an anti-PD nanostructure of interest, as well as of any surface optimization that overcomes it, will nanomedicines be able to achieve their therapeutic application in PD and similar neurodegenerative conditions. Only those nanotherapies that meet the requirements set out in Figure 3 (for the case of controlled dopamine release), that allow non-invasive delivery, and whose impact does not destabilize brain homeostasis at excessive detrimental levels as compared to the therapeutic effect should be approved for further clinical trials. Any other nanostructures should keep being optimized to meet these standards. In addition, the use of these nanotherapies could be complemented with the administration of nanosensors selective to DA and capable of measuring in real-time and at a cellular scale the variations in the concentrations and release kinetics of the neurotransmitter [308,309]. Thus, the impact of the applied nanostructures on the neurological disease-related processes of the patient could be monitored and, thus, the therapy adapted in a tailored manner with respect to the prognosis of the patient. Obviously, these nanosensors must satisfy, in turn, the requirements established for the nanostructures described in this work.

## 6. Conclusions

Given that the coordination and execution of motor, cognitive and limbic activities by the basal ganglia is so complex and regulated, it is not surprising that alterations in the physiological neurotransmission in DA result in symptoms as disabling as those of PD. The loss of proteostasis in dopaminergic neurons, especially for α-syn (responsible for vesicle docking, vesicle fusion, and DA release), generates a vicious cycle of degeneration such that, even if the loss of DA is directly addressed and motor symptoms are “resolved”, the disease progressively continues until any dopaminergic restitution therapy becomes futile. In this scenario, nanomedicine gains special attention as a scientific-technological approach for the development of therapies that re-establish neurotransmission under normal conditions, as well as those that directly attack the molecular causes responsible for neuronal death and neurodegeneration. Thus, new nanocarriers (polymeric, lipidic, and metallic, among others) allow the controlled release of DA in its basal state, under physiological conditions, and in the regions of interest. Similarly, the study of nanozymes is generating new advances in the design and fabrication of catalytic structures with oxidoreductase-like properties for the elimination of oxidative stress, chaperone-like for the inhibition of protein aggregation, and protease-like for the selective proteolysis of misfolded proteins. However, as expected, the development of nanotherapies, either for nanocarriers or nanozymes, has limitations. The main reasons encompass (i) the nature of the BBB that restricts the passage of molecules from the bloodstream into the brain, which hinders the use of systemic therapies; (ii) the need for highly selective nanostructures whose mechanism of action (drug release or nanocatalysis) takes place exclusively at sites of need; and (iii) the safety concerns regarding the mechanisms of action of NPs and their potential accumulation. However, as described above, promising advances in NPs surface optimization suggest the possibility of developing selective and biocompatible nanotherapies that also permit delivery by non-invasive routes, such as intranasal administration. However, this will only be possible as research continues into the molecular neuropathology of PD, the development of delivery and catalytic nanocomposites, and surface optimization for selectivity and biocompatibility.

## Data Availability

Not applicable.
